# The correlation between intraocular pressure and choroidal microcirculation in patients with high myopia

**DOI:** 10.7150/ijms.113035

**Published:** 2025-06-20

**Authors:** Dongjie Song, Yutong Han, Zongchan Zhang, Jiayun Ge, Kuangqi Chen, Jianping Tong, Ye Shen

**Affiliations:** 1 Department of Ophthalmology, the Fourth Affiliated Hospital of School of Medicine, and International School of Medicine, International Institutes of Medicine, Zhejiang University, Yiwu, China, 322000.; 2 Department of Ophthalmology, The First Affiliated Hospital, Zhejiang University School of Medicine, Hangzhou, Zhejiang, China.; 3 Department of Ophthalmology, The First Affiliated Hospital of Dalian Medical University, Dalian, Liaoning, China.; 4 Eye Hospital of Shandong First Medical University (Shandong Eye Hospital), Eye Institute of Shandong First Medical University, State Key Laboratory Cultivation Base, Shandong Provincial Key Laboratory of Ophthalmology, Jinan, Shandong, China; School of Ophthalmology, Shandong First Medical University, Jinan, Shandong, China.; 5 Department of Ophthalmology and Visual Sciences, The Chinese University of Hong Kong, Hong Kong, China.

**Keywords:** high myopia, intraocular pressure, choroidal thickness, choroidal vascularity, swept-source optical coherence tomography angiography

## Abstract

**Background:** High myopia (HM) is a leading cause of visual impairment worldwide and the choroid plays a key role in HM progression. Intraocular pressure (IOP) may also be involved in HM development, while the relationship between IOP and choroidal microcirculation in HM patients remains poorly characterized.

**Purpose:** To investigate the correlation between IOP and choroidal microcirculation in HM individuals using swept-source optical coherence tomography angiography (SS-OCTA).

**Methods:** This cross-sectional observational study enrolled 118 eyes of 118 participants aged 18-30, comprising 64 HM eyes (axial lengths (AL) ≥ 26mm) and 54 mild-to-moderate myopic (MM) eyes (AL < 26 mm). Data on spherical equivalent (SE), AL and central corneal thickness (CCT) were collected. IOP was measured using a non-contact tonometer (NCT) and corrected with a formula to minimize the effect of CCT. Based on ETDRS classification, choroidal thickness (ChT) and vascular parameters including choriocapillaris layer vessel density (ChCVD), choroidal vascular index (CVI), choroidal vascular volume (CVV), choroidal stromal volume (CSV) and choroidal stromal density (CSI) were calculated from SS-OCTA.

**Results:** In this cohort, HM eyes exhibited higher corrected IOP (*P* < 0.05) and lower ChT, CVV and CSV across all grids compared to MM eyes (*P <* 0.001). Increased CSI and decreased CVI were also observed in most sectors of the HM eyes (*P* < 0.05). After adjusting for age, gender and AL, corrected IOP was correlated with SE, ChT, CVI and CSI in the subfoveal central and inner nasal grids among HM patients (*P* < 0.05). No significant correlation was found between AL and corrected IOP (*P* > 0.05).

**Conclusion:** In our population, compared to MM eyes, HM eyes showed higher IOP levels, which were negatively correlated with SE but not AL. Additionally, elevated IOP was observed to be associated with reduced choroidal thickness and vascularity in specific macular regions. The potential link between IOP and choroidal microcirculation may be involved in HM progression.

## Introduction

Myopia, a highly prevalent refractive disorder, has become a growing public health concern worldwide 1, 2. By 2050, it is projected that 4.758 billion people will suffer from myopia globally, including 938 million with high myopia (HM), accounting for approximately 9.8% of the world's population 3. HM is defined as a refractive error with spherical equivalent (SE) ≤ -6.00 diopter (D) or axial length (AL) ≥ 26 mm 4. Individuals with HM are at higher risk of fundus complications, such as retinal detachment and macular degeneration, resulting in permanent visual impairment 5. The scientific consensus highlights the pivotal role of myopia control strategies in preventing HM and its associated complications 6. Currently, although a large number of studies have been conducted, the precise pathogenic mechanisms underlying myopia remain elusive 7-9. Investigating the key factors contributing to its progression is essential for the effective prevention and management of HM.

Recently, intraocular pressure (IOP) has been recognized as a potential factor in myopia development 10. Accumulating evidence has demonstrated a strong correlation between HM and glaucoma 11-14. Chong *et al.* identified a bidirectional genetic causal link between myopia and primary open-angle glaucoma, primarily mediated by IOP through Mendelian randomization analysis, highlighting the vital role of IOP in myopia progression 15. Some researchers proposed that lowering IOP might represent a novel therapeutic strategy for managing HM. Elevated IOP may exert direct mechanical stress on the sclera, thereby decreasing its elasticity and inducing axial elongation 16, 17. Additionally, high IOP may promote extracellular matrix degradation via downregulating tissue inhibitor of metalloproteinase-1 (TIMP-1) and upregulating matrix metalloproteinase-2 (MMP-2), modulating scleral remodeling and increasing axial length 18-20. Conversely, several studies attribute increased IOP in HM to anterior chamber changes and reduced aqueous humor outflow during accommodation 21, 22. Clinical research on the relationship between IOP and HM remains controversial, likely due to the variations in age, myopia severity and IOP measurement methods across different studies 23-28. Hence, more in-depth exploration is warranted.

Previous studies have proposed that the choroidal thickness (ChT) is generally lower in myopic eyes than in emmetropic and hyperopic individuals, correlating with myopia severity 10, 29, 30. As a highly vascularized structure, the choroid supplies oxygen and nutrients to the outer retina and the inner sclera, serving as a key component in scleral remodeling and ocular growth 31. Histological changes within choroid precede those in the retina during HM advancement, underscoring its importance in assessing disease progression 32. Notably, choroid vascular filling is intimately related to ChT and primarily affected by intraocular perfusion pressure, with IOP acting as a major regulator. However, the relationship between IOP and microcirculation in myopia development has not yet been elucidated and calls for further clinical exploration.

Swept-source optical coherence tomography angiography (SS-OCTA) is an advanced imaging technique that enables non-invasive and high-resolution observation of microcirculatory structures in the ocular fundus 30. Therefore, based on SS-OCTA, this study aims to evaluate IOP and choroidal microcirculation differences in the macular region between patients with HM and mild to moderate myopia (MM), exploring their correlations to provide new insights for managing HM.

## Methods

### Subjects

In this cross-sectional, observational, single-center study, 130 eyes of 130 patients with myopia were recruited at the Eye Center of the First Affiliated Hospital of Zhejiang University School of Medicine from July 2023 to December 2024. The study was conducted in accordance with the tenets of the Helsinki Declaration and received approval from the Human Research Ethics Committee of the First Affiliated Hospital of Zhejiang University School of Medicine. Written informed consent was obtained from all participants prior to examinations.

The inclusion criteria for this study were as follows: (1) participants aged 18-30 years; (2) SE ≤ -0.5 D; (3) IOP within the range of 10-21 mmHg after correction formula calculation; (4) high level of cooperation and adherence. The exclusion criteria included (1) diagnosed ocular diseases such as keratoconus, cataract, glaucoma and fundus disorders including retinal vasculitis, retinoschisis, macular degeneration, posterior staphyloma (PS), etc.; (2) known systemic diseases such as hypertension, diabetes, autoimmune diseases or systemic connective tissue diseases; (3) previous history of ocular surgery or trauma; (4) best-corrected visual acuity (BCVA) < 20/25; (5) inability to cooperate with various examinations or poor OCTA image quality.

Finally, healthy myopic individuals who had no significant history of ocular or systemic diseases and exhibited no typical fundus complications were enrolled. The subjects were further divided into two groups based on the degree of myopia: those with an AL ≥ 26 mm were defined as HM group, whereas the control group consisted of MM individuals with an AL<26 mm.

### Ophthalmic examinations

#### Routine ocular examinations

All participants underwent comprehensive ophthalmic examinations. Anterior segment examinations were performed using a slit-lamp biomicroscope (Haag-Streit BM 900, Köniz, Switzerland). Ocular fundus examinations were conducted with indirect ophthalmoscopy lenses (OI-STDM-LR, Ocular, USA) and an ultra-wide field scan laser ophthalmoscope (Daytona, Nikon, Japan). Refractive error and BCVA under cycloplegia were assessed by experienced optometrists using an auto-refractometer (RC5000, Tomey, Japan) and a comprehensive refractometer (RT-600, Nidek, Japan). The SE was calculated as spherical error + (1/2 × cylindrical error). AL and central corneal thickness (CCT) values were measured using an ophthalmic optical biometer (OA2000, Tomey, Japan), with the average values calculated from three repeated measurements.

#### IOP measurement and correction

IOP was measured using a non-contact tonometer (NCT) (TX 20, Canon, Japan), and the mean value of three consecutive measurements was recorded. Considering the potential influence of CCT on IOP values by NCT, we applied an IOP correction formula proposed by Zhongshan Eye Center: IOP _corrected_ = IOP _measured_ + (520 - CCT) / 43.48, which was derived from the data collected in the Chinese population 33.

#### OCTA examination and imaging analysis

**Figure 1:**
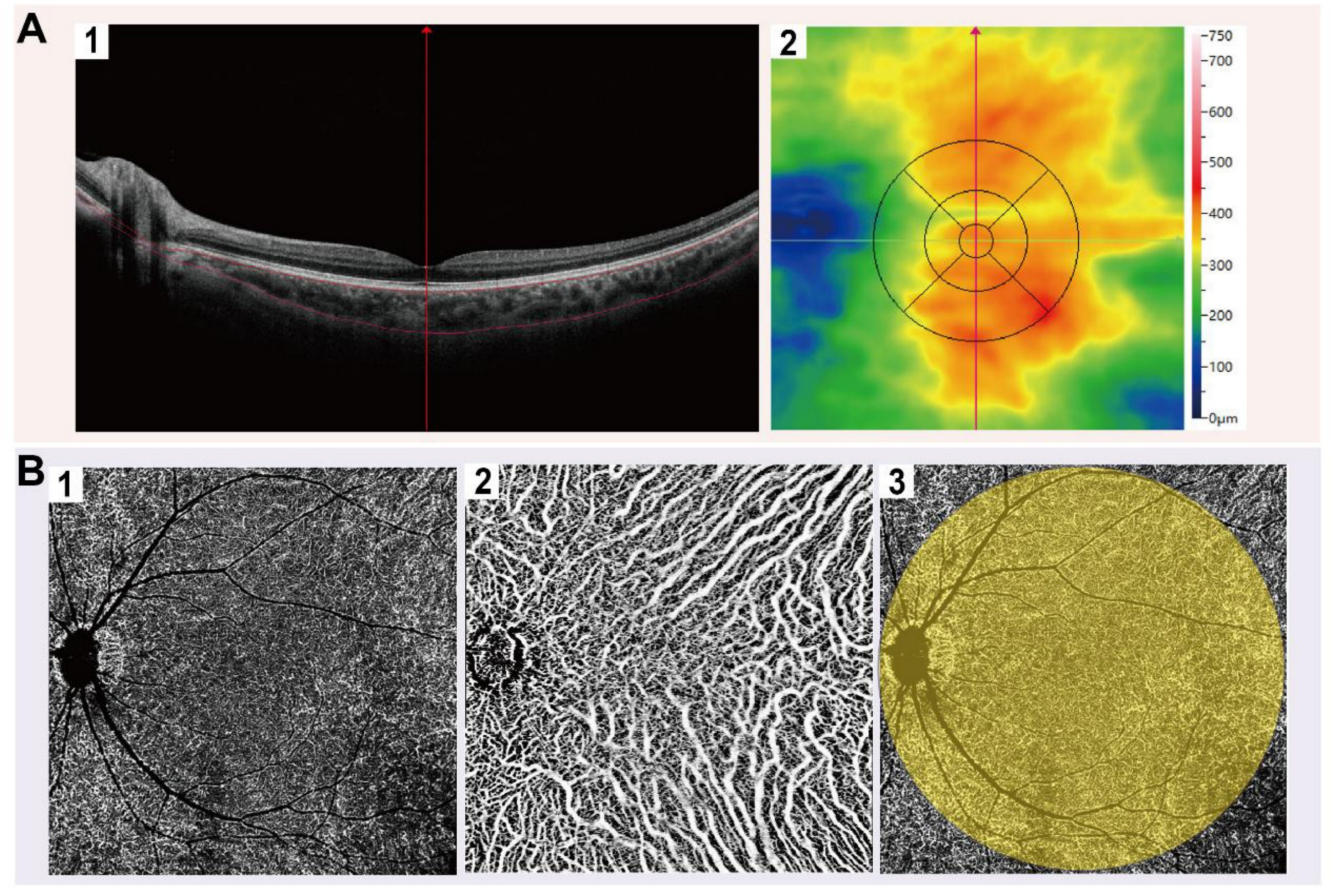
** Representative OCTA images of choroid.** (A) Representative macular images under En-face mode by OCTA. (A1) The distance from the Bruch's membrane to the lower boundary of the choroid is defined as ChT; (A2) ChT in ETDRS grids. (B) Representative choroidal vascular images by OCTA. (B1) OCTA images of ChC; (B2) OCTA images of ChV; (B3) Illustration of ChCVD.

We performed OCTA imaging using the SS-OCTA equipment (BM-400 K BMizar, TowardPi Medical Technology Co., Ltd., Beijing, China), which features a laser wavelength of 1060 nm, an acquisition rate of 400,000 A-scans per second, a bandwidth of 100 nm, and axial and transversal resolutions of 3.8 μm and 10 μm in tissue, respectively. Prior to the OCTA examination, patients were instructed to remain in a dimly lit environment for 20 minutes to ensure pupil dilation. The macular region was scanned with a 12 × 12 mm rectangular pattern incorporating motion correction, after precisely aligning the examination frame with the fovea and adjusting the focus to obtain a clear and well-illuminated fundus image. In en-face mode, choroidal images were initially segmented by the built-in automatic layering algorithm, and the obtained segmentation results were subsequently reviewed and adjusted as needed by an experienced ophthalmologist. The distance from Bruch's membrane to the lower boundary of the choroid was obtained as ChT** (Figure 1A)**.

**Figure 2:**
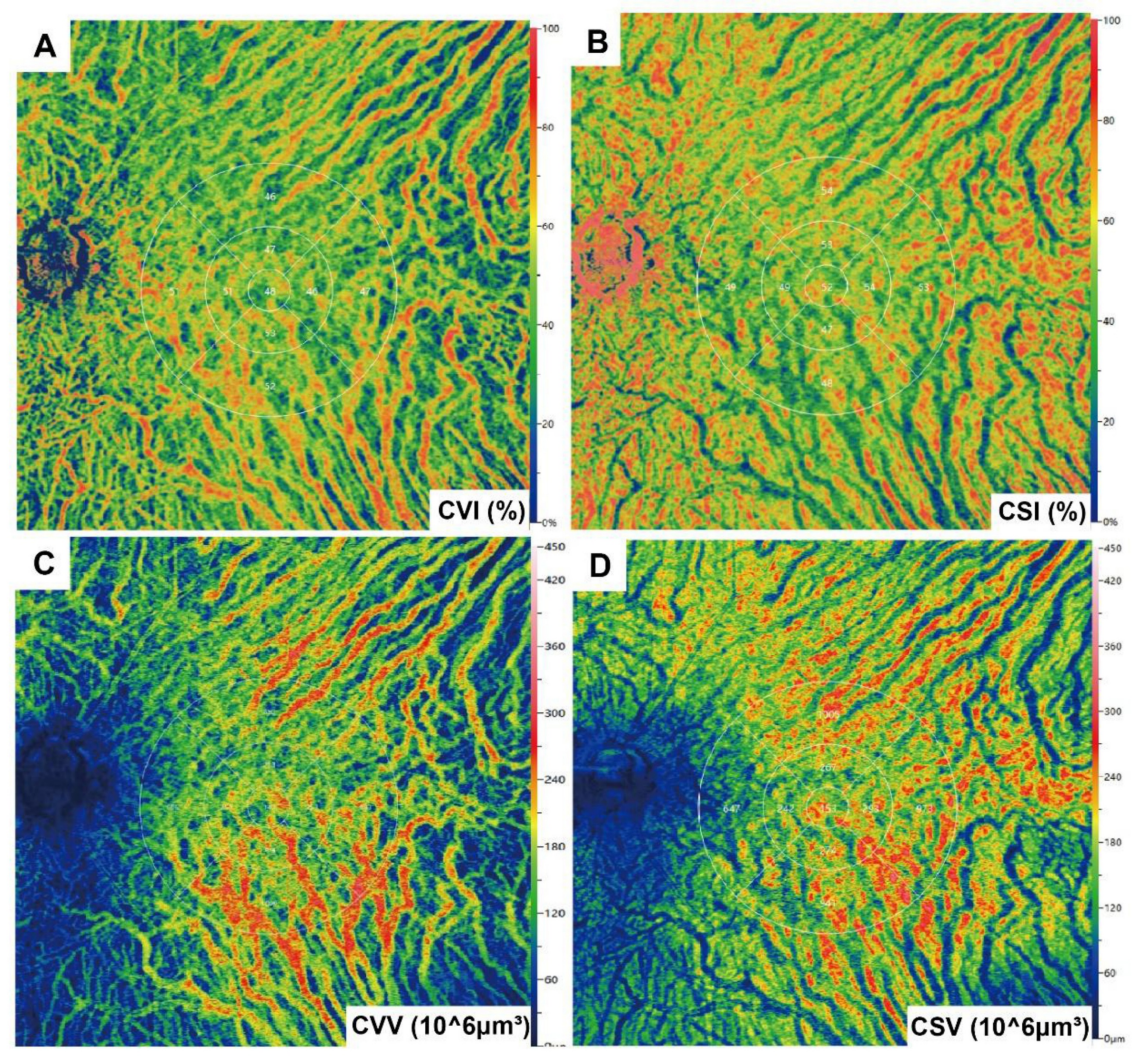
** Illustration of choroidal vascularity analysis.** CVI. (B) CSI. (C) CVV. (D) CSV.

Images meeting quality thresholds (with a score of 10) then underwent analysis using built-in angiography analysis software that employs the algorithm of higher-order moments amplitude-decorrelation angiography to evaluate choroidal microcirculation parameters 34, 35. The choriocapillaris layer (ChC) spans from Bruch's membrane to 29 µm beneath the membrane, while the choroidal vessel layer (ChV) extends from 29 µm below Bruch's membrane to the sclera. Choriocapillaris layer vessel density (ChCVD) is defined as the percentage of vascularized area within a circular region centered on the macular fovea with a diameter of 6 mm at the ChC layer, as determined by binarized image analysis **(Figure 1B)**. Subsequently, we conducted stereoscopic quantification of the ChV layer using the built-in algorithm to obtain relevant choroidal vascular parameters** (Figure 2)**: The choroidal vessel volume index (CVI) is calculated as the ratio of choroid vessel volume to the total choroid volume within a designated three-dimensional region, equivalent to a 3D vessel density, and expressed in percentage units. A higher CVI value frequently indicates greater vessel density. The choroidal vessel volume (CVV) is defined as choroid vessel volume per unit area (volume/area), and expressed in microns. A larger CVV value tends to indicate a greater number of vessels. The choroidal stroma volume index (CSI) is defined as the ratio of choroid stroma volume to choroid volume within a designated 3D region, and expressed in percentage units. The choroidal stroma volume (CSV) is defined as choroid stroma volume per unit area (volume/area), expressed in microns.

According to the Early Treatment Diabetic Retinopathy Study (ETDRS) classification, the macula centered on the fovea is divided into three concentric regions: the central 1 mm is defined as the fovea region, the annular area between 1-3 mm is defined as the parafovea region, and the annular area between 3-6 mm area is defined as the perifovea region. The parafovea and perifovea regions are further subdivided into four quadrants (superior, inferior, nasal, and temporal) 26. CT and vascular parameters were recorded for all nine regions to provide a more accurate and detailed description of the macular choroidal microcirculation characteristics.

To minimize the influence of circadian rhythm and ensure measurement consistency of the results, both the IOP and OCTA examinations were performed by the same experienced examiner between 14:00 and 16:00.

### Statistical analysis

Given the strong correlation between the right and the left eyes, the left eyes were selected as experimental subjects. All data were statistically analyzed using IBM SPSS Statistics 28.0 (IBM, Armonk, NY, USA). Descriptive statistics were presented as means ± standard deviations (SDs), and categorical variables were expressed as numbers and proportions. The Shapiro-Wilk (S-W) test was used to assess the normality of quantitative data. For normally distributed data with homogeneity of variance, independent samples t-test was applied for comparisons; for non-normally distributed data, a non-parametric rank sum test was used. Bonferroni correction was performed to adjust for multiple comparisons. For correlation analysis, Pearson's correlation was used for data with normal distribution while Spearman's rank correlation was applied for non-normal distributed data. Linear regression was conducted to evaluate the association between IOP and each outcome variable. *P <* 0.05 was considered statistically significant.

## Results

### Demographics

**Figure 3:**
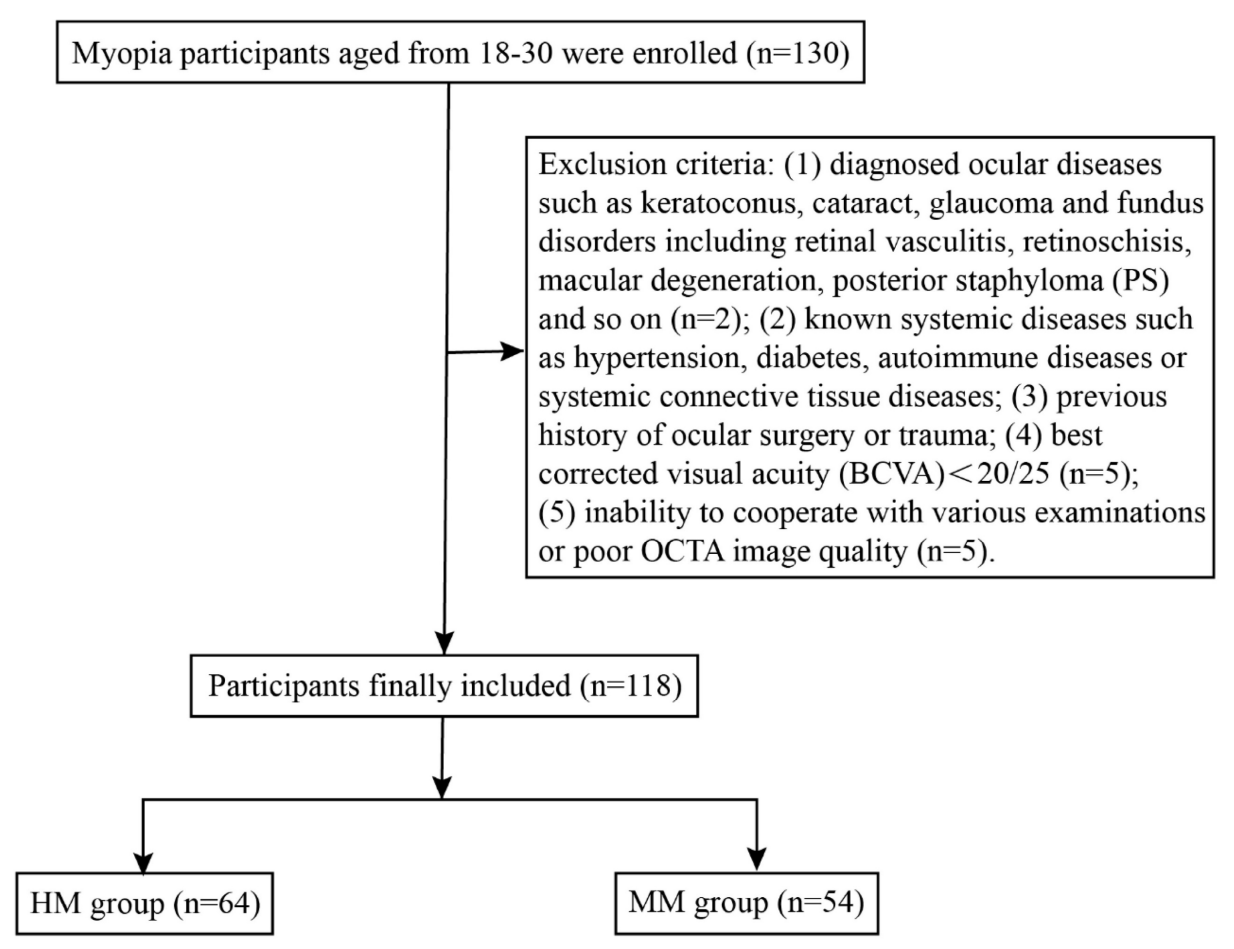
The workflow of participant selection for the study.

A total of 130 participants aged between 18 and 30 were successfully recruited and underwent detailed ophthalmic examinations, including OCTA. Among them, 12 participants were excluded due to asymptomatic retinoschisis (n = 1), retinoschisis combined with PS (n = 1), BCVA < 20/25 (n = 5) or sub-optimal OCTA image quality (n = 5). Ultimately, 118 eyes from 118 subjects (90.77%) were included in the analysis and categorized into two groups: the HM group (64 eyes, 64 subjects) and MM group (54 eyes, 54 subjects) **(Figure 3)**. The biometric parameters including age, gender, SE, AL and corrected IOP, did not differ significantly between the included participants and those excluded from the analysis** (Table 1)**. Demographic data of the included cohort are presented in **Table 2**. No significant differences were observed in age or gender between the two groups (*P* > 0.05), while AL and SE differed significantly between the HM and MM groups (*P* < 0.001). The mean AL was 27.00 ± 0.73 mm in the HM group and 25.10 ± 0.68 mm in the MM group. Notably, corrected IOP was significantly higher in the HM group than in the MM group (15.18 ± 2.10 mmHg vs. 14.24 ± 1.79 mmHg;* P* < 0.05) **(Figure S1)**.

### Measurements of choroidal parameters

**Figure 4:**
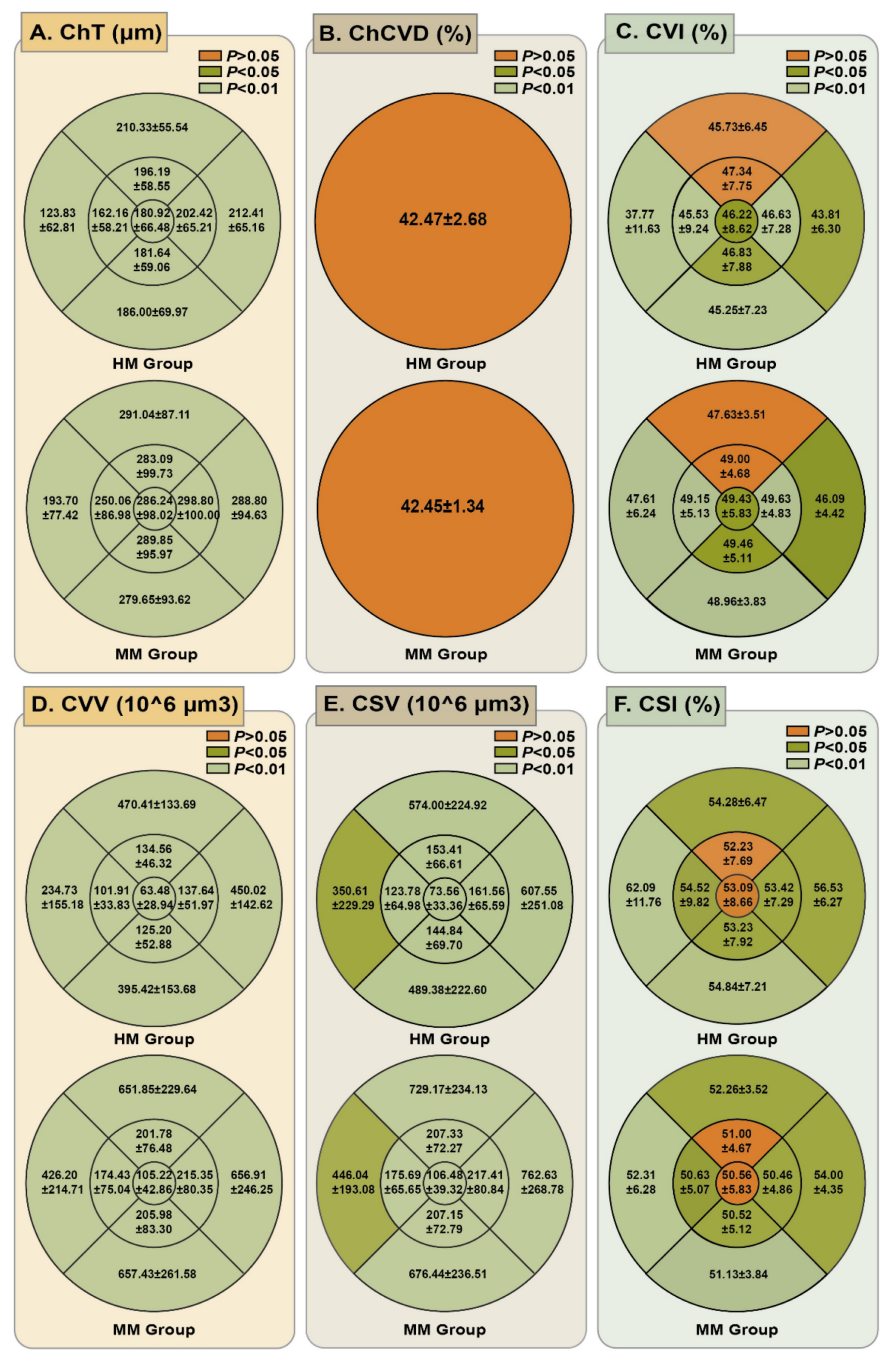
**Comparison of mean ChT and choroidal vascular parameters in different ETDRS grid sectors.** (A) Comparison of mean ChT in different ETDRS grids between HM group and MM group. (B) Comparison of ChCVD in a radius of 6 mm of the macular region between HM group and MM group. (C) Comparison of mean CVI in different ETDRS grids between HM group and MM group. (D) Comparison of mean CVV in different ETDRS grids between HM group and MM group. (E) Comparison of mean CSV in different ETDRS grids between HM group and MM group. (F) Comparison of mean CSI in different ETDRS grids between HM group and MM group.

We obtained the mean choroidal microcirculation parameters in all ETDRS grid sectors, as detailed in** Figure 4 (Table S1)**. Quantitative analysis revealed significantly reduced ChT in HM eyes compared to MM controls across all analyzed sectors (*P <* 0.001). The choroidal vessel density was also compared for all ETDRS grids. For the ChC layer, the ChCVD was 42.47 ± 2.68% in the HM group and 42.45 ± 1.34% in the MM group, showing no significant difference (*P* > 0.05). However, a statistically significant difference was observed in the ChV layer of the choroid. The CVI in the HM group was significantly lower than that in the MM group in all the grids except for the inner and the outer superior zones (*P* < 0.05). Compared with the MM group, the HM group demonstrated significantly lower CVV values in all sectors and decreased CSV in most sectors except for the outer nasal and temporal zones (*P* < 0.001). Conversely, patients with HM had a significantly higher CSI in all nine sectors except for the subfoveal central and inner superior sector (*P* < 0.05), compared to those in MM group.

### Correlation between IOP, myopia degree and choroidal microcirculation parameters

As shown in **Table 3**, a negative correlation was observed between corrected IOP and SE in HM eyes (r = -0.252, *P* = 0.045), while no significant correlation was found between corrected IOP and AL in either the HM group or the MM group. Furthermore, corrected IOP was significantly correlated with ChT in the subfoveal central (r = -0.312, *P* = 0.012), inner inferior (r = -0.268, *P* = 0.032), and inner nasal (r = -0.306, *P* = 0.014) grids in HM eyes. Additionally, our study demonstrated that CVI was negatively correlated with corrected IOP in the subfoveal central (r = -0.329, *P* = 0.008) and inner nasal (r = -0.300, *P* = 0.016) zones in HM eyes, whereas CSI exhibited an opposite trend (r = 0.250, *P* = 0.047 in the subfoveal center and r = 0.354, *P* = 0.004 in the inner nasal grids). Moreover, a negative correlation was observed between CVV and corrected IOP in the inner nasal grid (r = -0.256, *P* = 0.041) in the HM group, while in the MM group, a positive correlation was noted in the inner inferior (r = 0.324, *P* = 0.017) and outer inferior (r = 0.282, *P* = 0.039) sectors.

### Multiple regression analysis of IOP _corrected_, myopia degree and choroidal microcirculation parameters in HMs

We performed multivariate regression analysis, adjusting for age, gender, and AL, to explore the correlations between corrected IOP and other factors. The results are summarized in **Table 4**. After adjusting for age and gender (Model 2), corrected IOP in HMs was found to be significantly associated with ChT, CVI and CSI in both the subfoveal central and inner nasal grids as well as with ChT in the inner inferior grids.

In Model 3, after further adjustment for AL, these correlations generally remained significant. However, the association between inner inferior ChT and corrected IOP was no longer significant after adjustment.

## Discussion

Our results indicated a possible correlation between corrected IOP, SE, and choroidal microcirculation parameters in individuals with HM. We observed that corrected IOP was significantly higher in HM eyes compared to MM eyes. After adjusting for age, gender and AL, corrected IOP was negatively correlated with SE in HM patients in our study. Moreover, SS-OCTA analysis further revealed positive correlations between higher corrected IOP and increased ChT and CVI, as well as decreased CSI, particularly in the subfoveal central and inner nasal regions of HM patients. Further studies involving larger populations are warranted to validate these findings.

Currently, evidence regarding the correlation between IOP and myopia remains inconclusive. Nomura *et al.* reported significantly higher IOP levels in HM patients compared to non-HM individuals, while other studies found no significant correlation between IOP and myopia severity 36-40. Given that previous clinical investigations have consistently indicated that IOP tends to decline with age, we hypothesized that these discrepancies might arise from variations in subject age ranges and differences in IOP measurement methods 41-43. Therefore, in our study, 118 young myopic patients were included, with no significant age difference between groups to minimize age-related IOP variability. In addition, we used NCT with a correction formula to account for CCT effects on IOP measurement 33. The mean corrected IOP in the HM group was significantly higher than that in the MM group, partially supporting the findings of Nomura *et al.* and suggesting that IOP may be involved in myopia progression.

Our study also revealed a significantly negative correlation between corrected IOP and SE in HM patients, which is consistent with prior reports by Li *et al.* 39, 44, 45. We further conducted regression analysis which showed that corrected IOP was an independent factor influencing SE rather than AL, suggesting high IOP might affect HM advancement through mechanisms beyond axial elongation. We speculate that such dissociation may be attributed to ocular biomechanical alterations in HM eyes as well as the disparity of the underlying representation of SE and AL 46, 47. During the development of HM, the sclera undergoes extracellular matrix remodeling and exhibits increased extensibility and reduced stiffness, which may decouple the mechanical effects of IOP from axial elongation 48, 49. Yuan *et al.* reported that high myopic eyes exhibited a significantly lower scleral elastic modulus in both the macular and peripapillary regions compared to low myopic eyes 50. After being subjected to the same ocular pressure to increase IOP, researchers have observed that patients with low myopia recover significantly faster compared to those with HM 9. Thus, HM eyes with weakened scleral rigidity may demonstrate decreased resistance to IOP, allowing axial elongation to progress independently of IOP levels. Moreover, it has been reported that topical IOP-lowering agents could reduce myopia progression without affecting AL in the guinea pig form-deprivation model 51. Considering that SE reflects composite ocular parameters involving corneal curvature, lens thickness and vitreous chamber depth, we speculate that elevated IOP may alter corneal curvature by redistributing stress across the corneal stroma or induce changes of lens form, thereby contributing to HM development 40, 52. Furthermore, our study has observed that higher IOP in HM eyes is correlated with decreased choroidal blood flow in specific regions, potentially interfering with normal axial growth via mechanisms like scleral hypoxia and remodeling, and this will be discussed later. Of course, these hypotheses warrant validation through more systematic and rigorous experimental investigations.

Additionally, other studies have drawn differing conclusions on IOP-AL and IOP-SE association. Xu *et al.* identified a positive correlation between AL and IOP in extremely HM patients and a negative correlation between SE and IOP specifically in patients with SE ranging from -12 D to -15 D 53. While some studies have proposed that higher IOP correlates with slower axial growth in children with progressive myopia 28, 54. The variability of different studies may be attributed to the possible heterogeneity in the age of the subjects, the range of refractive power, and the methods of IOP measurement in different studies. Although the relationship between IOP and myopia remains undefined, the role of IOP in myopia progression should not be overlooked. Future studies ought to delve deeper into the direct impact of IOP on ocular biomechanics and conduct longitudinal assessments involving larger populations under standardized conditions.

Recently, the critical role of the choroid in myopia development has gained escalating attention. Our study found significantly thinner ChT in the central fovea and surrounding 6 mm grids in HM patients compared to MM patients, consistent with previous studies 32, 55. Additionally, ChT in the subfoveal, inner inferior, and inner nasal grids of HM individuals exhibited a negative correlation with corrected IOP. Preceding research has also indicated that IOP may contribute to the regulation of ChT. Wang *et al.* reported that after dark room tests induced a sharp increase in IOP, subfoveal ChT became thinner, with the extent positively correlated with baseline IOP levels 56. Furthermore, glaucoma patients using long-term IOP-lowering medications showed increased ChT 57. Mechanically, the choroid can modulate retinal position by altering its thickness in response to optical defocus signals, thus contributing to the regulation of axial elongation and myopia progression 58, 59. Based on these findings, we speculate that modulating IOP to adjust ChT could represent a potential strategy for preventing and controlling HM.

Given that the choroid is highly vascularized and its thickness is significantly influenced by vascular filling, we hypothesize that IOP may regulate ChT through modulation of choroidal blood flow. Consequently, we investigated the relationship between IOP and choroidal microcirculation parameters in HM patients. No statistically significant difference in ChCVD within a 6-mm radius of the macula region was found between HM and MM patients at the choroidal capillary layer, consistent with Mo *et al.*'s findings 60. However, several studies have reported significantly lower ChCVD in the macular region of HM eyes compared to MM eyes, correlating with myopia severity 26, 61. Cheng *et al.* noted increased choroidal capillary defect rates specifically in the perifoveal region during axial elongation in HM patients, with no significant changes observed in other regions 62. Since choroidal capillaries in different areas of the macular region are affected by HM to varying degrees, the disparities among studies may be ascribed to differences in observation scope. Therefore, in-depth exploration of the effects of IOP on choroidal capillaries across different fundus areas in HM eyes should be conducted to draw more reliable conclusions.

We employed three-dimensional quantitative indicators, including CVI, CVV, CSV, and CSI, to characterize the state of choroidal macrovasculature, providing a more precise and comprehensive assessment of choroidal structure compared to two-dimensional measurement 30. Our study showed that CVI, CVV and CSV were significantly reduced in HM eyes, while CSI was significantly increased across all analyzed grids, consistent with previous reports 6, 13, 26. Moreover, our findings indicated that in HM patients, elevated IOP was significantly correlated with reduced choroidal vascular density and increased stromal density in the subfoveal and inner nasal grids, whereas no significant differences were noted in vascular or stromal distribution in other areas. Given the cross-sectional nature of this study, it was not feasible to establish a causal relationship among IOP, choroidal microcirculation and HM development at present 63. Nevertheless, by incorporating the regional correlation between IOP and choroidal microcirculation identified in our present study with prevailing theories regarding the mechanisms underlying HM progression, it is possible to construct a speculative mechanism linking IOP to HM development. On the one hand, from the perspective of scleral hypoxia theory: elevated IOP may lead to decreased choroidal vascular density in the subfoveal and inner nasal regions, resulting in insufficient oxygen supply to the sclera 64, 65. Subsequently, the scleral hypoxia microenvironment may further induce scleral remodeling, making the posterior pole of the eye more prone to expansion and characteristic fundus changes like peripapillary atrophy (PPA) in the temporal region, ultimately promoting HM advancement 65. On the other hand, from the perspective of defocus signals theory: previous studies have shown that the nasal choroid exhibits greater sensitivity to myopic defocus signals compared to other regions, while the temporal choroid responds better to hyperopic defocus signals, which correlates with choroidal blood flow distribution 66, 67. As IOP increases, choroidal vascular perfusion may decrease in the subfoveal and inner nasal grids, potentially diminishing the sensitivity of nasal choroid to myopic defocus signals while increasing susceptibility to axial elongation, thereby facilitating HM development. However, it should be emphasized that the above speculations and hypotheses still require further validation through experimental research and large-scale longitudinal cohort studies.

There are still some limitations in this study. Firstly, it included a relatively small sample size of 118 patients with myopia and did not involve emmetropic individuals due to recruitment challenges, which may introduce sampling biases and limit the generalizability of our findings. As a cross-sectional study, it lacks longitudinal evaluation of the long-term effects of IOP on choroidal microcirculation, thereby restricting its clinical applicability. Although we applied a CCT-adjusted formula based on Chinese populations for IOP correction, its validity for HM eyes with altered corneal biomechanics such as corneal hysteresis remains unconfirmed. Prospective studies incorporating multimodal tonometry like Goldmann applanation tonometry (GAT) or dynamic contour tonometry are required to confirm our observations in HM cohorts 68. Moreover, we did not consider subclinical PS in HM eyes and future studies could incorporate ultra-widefield OCTA and color Doppler imaging technology to better assess potential PS on regional choroidal microcirculation.

Nevertheless, our results have indicated that the possible association between IOP and choroidal microcirculation may be involved in the occurrence and progression of HM patients. Even though IOP levels in this study were within the normal clinical range, relatively higher IOP was still observed to damage choroidal microcirculation in the macular region, with the extent of damage correlating closely with myopia severity. Therefore, we recommend that clinicians closely monitor IOP in HM patients and consider establishing a standardized IOP range specific for HM eyes to improve management precision. Admittedly, while the interaction between IOP and HM requires further investigation, understanding their correlation with choroidal microcirculation could enhance insights into HM progression and inform preventive strategies.

## Conclusion

In conclusion, our findings have indicated that compared to MM individuals, those with HM may exhibit higher levels of IOP. Moreover, as IOP increased, the choroidal vascularity of HM eyes tended to be sparser while the stromal structure appeared denser in the central fovea and nasal parafovea. This potential association between IOP and choroidal microcirculation in specific macular regions may be involved in the development of HM. Further research involving larger sample sizes and longitudinal assessments is essential to elucidate the effects of IOP on choroidal microcirculation and its implications for the physiological and pathological mechanisms underlying myopia.

## Supplementary Material

Supplementary figure and table.

## Figures and Tables

**Table 1:** Demographic characteristics of the participants and the non-participants

	Included participants (n = 118)	Excluded participants (n = 12)	*P* value
Gender (n, %)			
Male	60 (50.8)	5 (41.7)	0.763
Female	58 (49.2)	7 (58.3)	
Age (y), mean±SD	24.71±3.16	23.92±2.61	0.401
AL (mm), mean±SD	26.13±1.19	26.57±0.70	0.216
SE (D), mean±SD	-7.48±3.17	-8.06±1.91	0.363
IOP _corrected_ (mmHg), mean±SD	14.75±2.01	14.18±2.23	0.353

**Table 2:** Demographic characteristics of the study cohort

	HM (n = 64)	MM (n = 54)	*P* value
Gender (n, %)			
Male	33 (51.6)	27 (50)	0.866
Female	31(48.4)	27 (50)	
Age (y), mean±SD	24.28±3.28	25.22±2.96	0.107
AL (mm), mean±SD	27.00±0.73	25.10±0.68	<0.001
SE (D), mean±SD	-9.39±2.15	-5.22±2.66	<0.001
IOP _corrected_ (mmHg), mean±SD	15.18±2.10	14.24±1.79	0.011

**Table 3:** Correlation between IOP _corrected_ and myopia and choroidal circulation parameters

	HM		MM	
	r	*P* value	r	*P* value
SE	-0.252	0.045	0.154	0.265
AL	-0.042	0.741	-0.133	0.336
ChT				
Subfoveal central	-0.312	0.012	0.216	0.116
Inner superior	0.034	0.791	0.087	0.530
Inner inferior	-0.268	0.032	0.259	0.058
Inner nasal	-0.306	0.014	0.142	0.307
Inner temporal	0.018	0.888	0.180	0.193
Outer superior	0.072	0.573	0.011	0.940
Outer inferior	0.014	0.915	0.221	0.109
Outer nasal	0.105	0.408	0.089	0.521
Outer temporal	-0.026	0.837	0.189	0.170
ChCVD	-0.010	0.935	-0.073	0.601
CVI				
Subfoveal central	-0.329	0.008	0.070	0.616
Inner superior	-0.106	0.407	-0.002	0.988
Inner inferior	-0.146	0.251	0.258	0.060
Inner nasal	-0.300	0.016	0.142	0.304
Inner temporal	-0.034	0.787	-0.032	0.819
Outer superior	-0.061	0.629	0.110	0.428
Outer inferior	-0.106	0.405	0.187	0.177
Outer nasal	-0.032	0.800	0.198	0.151
Outer temporal	-0.019	0.881	-0.010	0.943
CVV				
Subfoveal central	0.085	0.507	0.240	0.081
Inner superior	0.025	0.845	0.044	0.755
Inner inferior	0.014	0.912	0.324	0.017
Inner nasal	-0.256	0.041	0.193	0.161
Inner temporal	-0.005	0.968	0.211	0.125
Outer superior	0.065	0.612	0.082	0.556
Outer inferior	-0.081	0.523	0.282	0.039
Outer nasal	0.155	0.220	0.104	0.455
Outer temporal	-0.067	0.602	0.238	0.083
CSV				
Subfoveal central	0.079	0.533	0.133	0.338
Inner superior	0.017	0.895	0.066	0.637
Inner inferior	0.026	0.836	0.159	0.252
Inner nasal	0.062	0.626	0.075	0.592
Inner temporal	-0.091	0.473	0.171	0.216
Outer superior	0.038	0.768	-0.001	0.996
Outer inferior	0.050	0.695	0.150	0.279
Outer nasal	0.044	0.729	0.110	0.428
Outer temporal	-0.024	0.848	0.174	0.208
CSI				
Subfoveal central	0.250	0.047	-0.067	0.629
Inner superior	0.035	0.781	0.001	0.993
Inner inferior	0.086	0.497	-0.266	0.052
Inner nasal	0.354	0.004	-0.177	0.199
Inner temporal	0.041	0.747	0.029	0.835
Outer superior	0.052	0.684	-0.114	0.414
Outer inferior	0.112	0.380	-0.183	0.184
Outer nasal	0.027	0.834	-0.189	0.172
Outer temporal	0.033	0.793	0.011	0.145

**Table 4:** Multiple linear regression analyses of the effects of IOP _corrected_ on SE and choroidal vascular parameters in HMs

Outcomes	Model 1		Model 2		Model 3	
	β (95% CI)	*P* value	β (95% CI)	*P* value	β (95% CI)	*P* value
SE	-0.32 (-0.56, -0.08)	0.011	-0.32 (-0.56, -0.08)	0.012	-0.28 (-0.48, -0.09)	0.006
Subfoveal central CVI	-2.02 (-2.91, -1.13)	<0.001	-1.99 (-2.88, -1.09)	<0.001	-1.93 (-2.81, -1.05)	<0.001
Inner nasal CVI	-1.65 (-2.66, -0.64)	0.002	-1.53 (-2.51, -0.55)	0.003	-1.50 (-2.47, -0.52)	0.004
Inner nasal CVV	-5.60 (-9.35, -1.84)	0.005	-5.50 (-9.30, -1.70)	0.006	-5.35 (-9.14, -1.56)	0.008
Subfoveal central CSI	1.32 (0.35, 2.29)	0.010	1.28 (0.30, 2.26)	0.013	1.26 (0.27, 2.25)	0.015
Inner nasal CSI	1.94 (0.89, 3.00)	<0.001	1.85 (0.81, 2.90)	<0.001	1.84(0.78, 2.90)	0.001
Subfoveal central ChT	-11.79 (-19.10, -4.49)	0.002	-11.42 (-18.79, -4.04)	0.004	-11.20 (-18.60, -3.80)	0.004
Inner inferior ChT	-7.38 (-15.84, 1.08)	0.090	-7.06 (-15.62, 1.50)	0.109	-2.25 (-9.23, -4.74)	0.530
Inner nasal ChT	-9.64 (-15.94, -3.34)	0.004	-9.73 (-16.11, -3.35)	0.004	-9.38(-15.66, -3.10)	0.005

Model 1, Crude; Model 2, adjusting for age and gender; Model 3 adjusting for age, gender and AL; CI: Confidence Interval

## Abbreviations

HM: high myopia
SE: spherical equivalent
D: diopter
AL: axial length
IOP: intraocular pressure
TIMP-1: tissue inhibitor of metalloproteinase-1
MMP-2: matrix metalloproteinase-2
ChT: choroidal thickness
SS-OCTA: swept-source optical coherence tomography angiography
MM: mild to moderate myopia
PS: posterior staphyloma
BCVA: best-corrected visual acuity
CCT: central corneal thickness
NCT: non-contact tonometer
ChC: choriocapillaris layer
ChV: choroidal vessel layer
ChCVD: choriocapillaris layer vessel density
CVI: choroidal vascular index
CVV: choroidal vascular volume
CSI: choroidal stromal density
CSV: choroidal stromal volume
ETDRS: Early Treatment Diabetic Retinopathy Study
SDs: standard deviations
S-W test: Shapiro-Wilk test
PPA: peripapillary atrophy
GAT: Goldmann applanation tonometry
