# Supplementary material for: The correlation between intraocular pressure and choroidal microcirculation in patients with high myopia
Source: Int J Med Sci. 2025 Jun 20;22(12):3032–43. doi: 10.7150/ijms.113035 (PMC12244010; doi:10.7150/ijms.113035)
Supplement: Supplementary file 1 — Supplementary figure and table. [file ijmsv22p3032s1.pdf]

Supplementary Materials

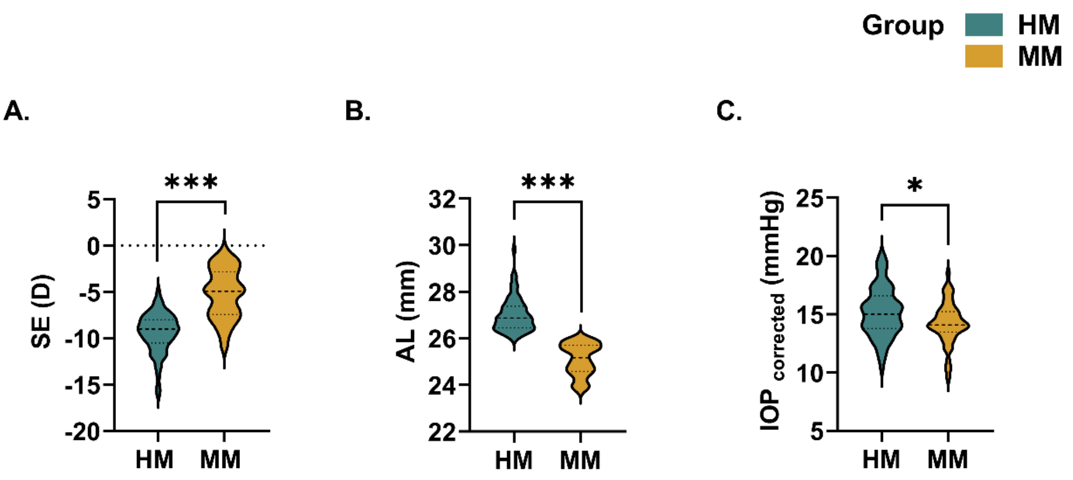

Figure S1 Comparison of SE, AL, and IOP $_{corrected}$ in HM and MM group

(A) Comparison of SE between HM group and MM group. (B) Comparison of AL between HM group and MM group. (C) Comparison of IOP $_{corrected}$ between HM group and MM group

$^{*}P<0.05$; $^{**}P<0.01$; $^{***}P<0.001$

Table S1 Mean ChT and choroidal vascular parameters in different ETDRS grid sectors

Choroidal parameters	HM	MM	*P* value*
ChT (μm)			
Subfoveal central	180.92±66.48	286.24±98.02	<0.001
Inner superior	196.19±58.55	283.09±99.73	<0.001
Inner inferior	181.64±59.06	289.85±95.97	<0.001
Inner nasal	162.16±58.21	250.06±86.98	<0.001
Inner temporal	202.42±65.21	298.80±100.00	<0.001
Outer superior	210.33±55.54	291.04±87.11	<0.001
Outer inferior	186.00±69.97	279.65±93.62	<0.001
Outer nasal	123.83±62.81	193.70±77.42	<0.001
Outer temporal	212.41±65.16	288.80±94.63	<0.001
ChCVD (%)	42.47±2.68	42.45±1.34	0.967
CVI (%)			
Subfoveal central	46.22±8.62	49.43±5.83	0.022
Inner superior	47.34±7.75	49.00±4.68	0.172
Inner inferior	46.83±7.88	49.46±5.11	0.037
Inner nasal	45.53±9.24	49.15±5.13	0.012
Inner temporal	46.63±7.28	49.63±4.83	0.011
Outer superior	45.73±6.45	47.63±3.51	0.056
Outer inferior	45.25±7.23	48.96±3.83	0.001
Outer nasal	37.77±11.63	47.61±6.24	<0.001
Outer temporal	43.81±6.30	46.09±4.42	0.027
CVV (10^6 μm^3)			
Subfoveal central	63.48±28.94	105.22±42.86	<0.001
Inner superior	134.56±46.32	201.78±76.48	<0.001
Inner inferior	125.20±52.88	205.98±83.30	<0.001
Inner nasal	101.91±33.83	174.43±75.04	<0.001
Inner temporal	137.64±51.97	215.35±80.35	<0.001
Outer superior	470.41±133.69	651.85±229.64	<0.001
Outer inferior	395.42±153.68	657.43±261.58	<0.001
Outer nasal	234.73±155.18	426.20±214.71	<0.001
Outer temporal	450.02±142.62	656.91±246.25	<0.001
CSV (10^6 μm^3)			
Subfoveal central	73.56±33.36	106.48±39.32	<0.001
Inner superior	153.41±66.61	207.33±72.27	<0.001

	HM	MM	*P* value*
CSV (10^6 μm^3)			
Inner inferior	144.84±69.70	207.15±72.79	<0.001
Inner nasal	123.78±64.98	175.69±65.65	<0.001
Inner temporal	161.56±65.59	217.41±80.84	<0.001
Outer superior	574.00±224.92	729.17±234.13	<0.001
Outer inferior	489.38±222.60	676.44±236.51	<0.001
Outer nasal	350.61±229.29	446.04±193.08	0.017
Outer temporal	607.55±251.08	762.63±268.78	0.002
CSI (%)			
Subfoveal central	53.09±8.66	50.56±5.83	0.07
Inner superior	52.23±7.69	51.00±4.67	0.305
Inner inferior	53.23±7.92	50.52±5.12	0.032
Inner nasal	54.52±9.82	50.63±5.07	0.010
Inner temporal	53.42±7.29	50.46±4.86	0.012
Outer superior	54.28±6.47	52.26±3.52	0.042
Outer inferior	54.84±7.21	51.13±3.84	0.001
Outer nasal	62.09±11.76	52.31±6.28	<0.001
Outer temporal	56.53±6.27	54.00±4.35	0.014

**P* values after Bonferroni's correction
